# Neuroprotective Potential of Thinned Peaches Extracts Obtained by Pressurized Liquid Extraction after Different Drying Processes

**DOI:** 10.3390/foods11162464

**Published:** 2022-08-16

**Authors:** Chongting Guo, Alberto Valdés, José David Sánchez-Martínez, Elena Ibáñez, Jinfeng Bi, Alejandro Cifuentes

**Affiliations:** 1 Institute of Food Science and Technology, Chinese Academy of Agricultural Sciences (CAAS), Beijing 100193, China; 2 Foodomics Laboratory, Instituto de Investigación en Ciencias de la Alimentación, CSIC-UAM, Nicolás Cabrera 9, 28049 Madrid, Spain; 3 Department of Food Science, Shenyang Agricultural University, Shenyang 110866, China

**Keywords:** blood–brain barrier, food by-products, neuroprotective activity, PLE, polyphenols, thinned peach fruits

## Abstract

Genetic, environmental and nutritional factors are suggested as primary factors of Alzheimer’s disease (AD), and secondary metabolites such as polyphenols present in thinned peaches are considered as good candidates for AD prevention. Thinned peaches are usually dried to avoid putrefaction, but the effects of the drying method and the extraction process on the polyphenol composition and the neuroprotective potential have never been addressed. In this work, a pressurized liquid extraction (PLE) method was optimized and applied to thinned peaches dried under different conditions, and their neuroprotective potential was evaluated in vitro. In addition, the PLE extracts were characterized via HPLC-Q-TOF-MS/MS, and a permeability assay was performed to evaluate the ability of the identified metabolites to cross the blood–brain barrier (BBB). The PLE extracts obtained from freeze-dried (FD) samples with 50% ethanol in water at 180 °C showed the best neuroprotective potential. Finally, among the 81 metabolites identified, isoferulic acid, 4-methyldaphnetin, coniferyl aldehyde and 3,4-dihydroxyacetophenone were found at higher concentrations in FD extracts. These metabolites are able to cross the BBB and are positively correlated with the neuroprotective potential, suggesting FD together with PLE extraction as the best combination to exploit the neuroprotective capacity of thinned peaches.

## 1. Introduction

Alzheimer’s disease (AD) is the most common neurodegenerative disease, and over 55 million people live with dementia worldwide [1]. The neuropathological features that identify AD are defined as neurotic plaques and neurofibrillary tangles, which are manifested by several symptoms such as cognitive impairment, delusions and hallucinations. Recent research studies have demonstrated that the increases in acetylcholinesterase (AChE) and butyrylcholinesterase (BChE) activity levels are the primary factors responsible for the progressive memory loss of AD patients [2,3]. Oxidative stress through the accumulation of reactive oxygen species (ROS) and reactive nitrogen species (RNS) and neuroinflammatory processes linked to the enzyme lipoxygenase (LOX) have also been suggested as leading causes of neurodegeneration [4,5], and a great number of secondary metabolites from plants have been described to have neuroprotective potential [6].

Thinned peaches are the young peaches that are manually removed from trees to ensure good quality and yield. This peach by-product has been shown to have an increased polyphenol content as compared to ripe peaches [7]. Approximately 1.25 million tons of thinned young peach are obtained in China every year, and drying the fruit is a necessary treatment to prevent putrefaction during storage [8]. However, the chemical structures of most polyphenols are unstable and easily affected by external conditions such as oxygen, temperature and UV radiation, meaning the different drying treatments can change the polyphenol composition of the fruit, affecting their biological activity. In this regard, different drying methods such as freeze drying (FD), natural drying (ND) and hot air drying (HAD) have been combined with ultrasound-assisted extraction (UAE) to explore the polyphenol composition of thinned peaches and their bioactivity [9,10]; however, these drying processes can also be combined with more advanced extraction methods, such as pressurized liquid extraction (PLE). This technology is characterized by its high extraction efficiency, safety and the use of solvents that are generally recognized as safe (ethanol or water) to extract bioactive compounds by using high pressures and temperatures above the boiling point and below the critical point [11]. In addition, it uses shorter times and less solvents than other extraction methods, such as UAE, with the extraction temperature and solvent composition being the most dominant factors.

The aim of the present work was to optimize and evaluate the in vitro neuroprotective potential of PLE extracts obtained from different dried thinned peach samples, to compare these extracts to UAE extracts and to characterize their polyphenol-derived metabolites using HPLC-Q-TOF-MS/MS. In addition, the neuroprotective potential of the identified metabolites was also evaluated through the study of their ability to cross the blood–brain barrier (BBB). The BBB represents a complex and dynamic barrier between the central nervous system (CNS) and the systemic circulation, and due to its restrictive and selective permeability, it limits the entrance of bioactive compounds to the brain parenchyma to exert their neuroprotective potential [12]. Among the different in vitro models, the parallel artificial membrane permeability assay for the BBB (PAMPA-BBB) assumes a high-throughput non-cell-based permeation test, which has been widely validated to study the rate of transcellular passive diffusion of the BBB [13].

## 2. Materials and Methods

### 2.1. Chemicals and Reagents

The Folin–Ciocalteu reagent was obtained from Merck (Darmstadt, Germany). Trizma hydrochloride (Tris-HCl), acetylcholinesterase (AChE), butyrylcholinesterase (BChE), naphthylethylene diamine dihydrochloride, sulphanilamide, acetylthiocholine iodide, linoleic acid, aluminum chloride, phosphoric acid, sodium carbonate, disodium phosphate, potassium phosphate, monopotassium phosphate, sodium nitroprusside dehydrate, fluorescein, gallic acid, quercetin, galantamine hydrobromide, ascorbic acid, *n*-dodecane, cholesterol, porcine polar brain lipid, a PAMPA-BBB 96-well donor plate (Catalog no MAIPNTR10) and a 96-well acceptor plate (Catalog no MATRNPS50) were purchased from Sigma-Aldrich (Madrid, Spain). The lipoxygenases from glycine max (soybean), 2,2-azobis(2-amidinopropane) dihydrochloride (AAPH) and 4-(amino-359 sulfonyl)-7-fluoro-2,1,3-benzoxadiazole (ABDF) were obtained from TCI Chemicals (Tokyo, Japan). The LC-MS-grade acetonitrile (ACN) was obtained from VWR Chemicals (Barcelona, Spain). The ultrapure water was obtained from a Millipore system (Billerica, MA, USA). The formic acid was purchased from Fisher Scientific (Waltham, MA, USA). The internal standard 12-[((cyclohexylamino)-carbonyl)amino]-dodecanoic acid (CUDA) was purchased from LabClinics (Ann Arbor, MI, USA). The isotope-labelled standard quercetin-d3, trans-cinnamic acid-d5 and reserpine-d9 were purchased from Toronto Research Chemical (Toronto, ON, Canada), and the hippuric acid-d5 was provided by Cambridge Isotope Laboratories, Inc. (Andover, MA, USA).

### 2.2. Sample Preparation

Thinned peach fruit of the “Zaoyu” variety were used as the raw materials. These fruit underwent cleaning, slicing (thickness of 1.0 mm) and drying, including with FD, ND and HAD at 50 °C (HAD50); HAD at 70 °C (HAD70); and HAD at 90 °C (HAD90). The FD was performed by freezing the samples at −80 °C for 24 h and then drying them in a lyophilizer for 36 h. The ND was performed at an average temperature of 30 °C. The ND, HAD50, HAD70 and HAD90 treatments lasted for 18 h at an air velocity of 1.0 m/s and a humidity of 8%. Thereafter, the dried thinned peaches were crushed into powder and stored in a freezer at −80 °C.

### 2.3. Ultrasonic-Assisted Extraction (UAE)

The UAE extraction of dried thinned peaches was performed according to our previous method [9]. Briefly, 1 g of different thinned peach powders was put in 20 mL solvent of 80% methanol in water (*v*/*v*) and extracted with the assistance of ultrasound (40 kHz) for 30 min at room temperature. The residue was re-extracted twice with the same solvent, and the supernatants were mixed, filtered and dried. Finally, the extracts were purified via solid-phase extraction.

### 2.4. Pressurized Liquid Extraction (PLE)

The PLE extraction of dried thinned peaches was carried out using an accelerated solvent extractor (ASE 200, Dionex, Sunnyvale, CA, USA) equipped with a solvent controller. The extraction was conducted according to a previous method [11]. Two factors were considered at three levels, namely the temperature (50, 115 and 180 °C) and solvent composition (ethanol at 0, 50% and 100% in Milli-Q water). The dried thinned peach sample (1 g) and sea sand (3 g) were mixed and placed into an 11 mL extraction cell. The extraction conditions were: time, 20 min; pressure, 10 MPa, heat-up time, 5 min; static extraction time, 5 min; flush volume, 60%; purge, N_2_ for 60 s. The extracts were allowed to stand in darkness and placed in a freezer at −20 °C. Finally, samples extracted with 100% ethanol were dried under nitrogen flow, the samples extracted with 50% ethanol in water were first dried under nitrogen flow and then freeze-dried and the samples extracted with water were directly freeze-dried. To optimize the extraction of compounds with neuroprotective potential, a response surface methodology (RSM) was performed by using a central composite design (CCD). The response variables studied were the total phenolic content (TPC), enzymatic inhibition activity (AChE and LOX) and antioxidant capacity (ROS), calculated as described below. Data for the experimental design and multi-response optimization were analyzed using Statgraphics Centurion XVI software (StatPoint Technologies, Inc., Warrenton, VA, USA). The analysis of variance (ANOVA), coefficient of determination (R^2^) of response surfaces, *p* values for the model, standardized Pareto charts, interaction plot and lack-of-fit testing were performed, accepting significance at *p* < 0.05 (see Tables S1–S6 and Figures S1 and S2, Supplementary Materials).

### 2.5. Extraction Yield, Total Phenolic Content (TPC) and Total Flavonoid Content (TFC)

The extraction yield is expressed as the percentage of the extract mass on a dry basis and the mass of initial thinned peach powder fed into the extraction cell. The TPC values of the different thinned peach extracts were assessed according to the Folin–Ciocalteu method [14], and the TFC values were evaluated according to a previously described method [11,15]. The TPC results are expressed as milligrams of gallic acid equivalent per gram of dried thinned peach extract (mg GAE/g), and the TFC results are expressed as milligrams of quercetin equivalent per gram of extract (mg QE/g). All experiments were performed in triplicate.

### 2.6. In Vitro Assays

#### 2.6.1. Anti-Cholinergic Activity

The AChE and BChE inhibitory activity levels of different thinned peach extracts were estimated according to the fluorescent enzyme kinetic method described by Sánchez-Martínez et al. (2021) [16]. In brief, 100 μL of extracts at different concentrations in 50% ethanol (*v*/*v*) were placed in a black 96-well plate, then 100 μL of buffer (150 mmol/L Tris-HCl, pH 8) and 25 μL of 0.8 U/mL enzyme (AChE or BChE in buffer) were added to each well. After incubating the mixtures for 10 min, 25 μL of 125 μmol/L ABDF in buffer and 50 μL of acetylthiocholine iodide were added to the mixture. Galantamine hydrobromide was used as the reference inhibitor and 50% ethanol was used as the control. The inhibition percentage was calculated according to Sánchez-Martínez et al. (2021) [16].

#### 2.6.2. LOX Inhibitory Activity

The LOX inhibitory activity was determined according to Whent et al. (2010) [17], with some modifications. Briefly, 100 μL of the extracts at different concentrations (100~1000 μg/mL) in 25% ethanol (*v*/*v*) was added to a black 96-well plate. Afterwards, 75 μL of 2 μmol/L fluorescein in buffer (150 mmol/L Tris-HCl, pH 9), 75 μL of 20.8 U/mL LOX in buffer and 100 μL of linoleic acid (in a concentration that equals to the KM value) in 25% ethanol were added to each well in sequence. Quercetin was used as a reference inhibitor and 25% ethanol was used as a control. The percentage of LOX inhibitory activity was calculated as described by Sánchez-Martínez et al. (2021) [16].

#### 2.6.3. ROS and RNS Scavenging Capacity

The ROS scavenging capacity was measured using the oxygen radical absorbance capacity (ORAC) assay described by Ou et al. (2001) [18]. Briefly, the reaction mixture contained 100 μL of samples at different concentrations in 10% ethanol (*v*/*v*), 100 μL of 590 mmol/L AAPH in 30 mmol/L phosphate buffer (pH 7.5), 100 μL of phosphate buffer and 25 μL of 10 μmol/L fluorescein in buffer. The absorbance was read at the 485 nm excitation spectrum and 528 nm emission spectrum, and recorded at 5 min intervals for 60 min at 37 °C. Ascorbic acid was used as the reference standard and 10% ethanol (*v*/*v*) was used as the control. The ROS scavenging capacity was calculated as described by Sánchez-Martínez et al. (2021) [16].

The RNS scavenging capacity was estimated by referring to the nitric oxide (NO) radical scavenging assay described by Ho et al. (2010) [19], with some modifications. In brief, 100 μL of the thinned peach extracts in 25% ethanol (*v*/*v*) was mixed with 50 μL of 5 mM sodium nitroprusside dehydrate in buffer (30 mmol/L phosphate buffer at pH 7.5) using a transparent 96-well plate. The mixtures were placed under white light at room temperature for 60 min. Afterwards, 100 μL of Griess reagent (500 mg sulphanilamide, 50 mg naphthylethylene diamine dihydrochloride and 1.25 mL of phosphoric acid in 48.5 mL of H2O) was added to each well. The mixtures were allowed to stand for 5 min and the absorbance was read at 546 nm. Ascorbic acid was used as the reference standard and the NO scavenging capacity was calculated as described by Sánchez-Martínez et al. (2021) [16].

### 2.7. Blood–Brain Barrier (BBB)

The parallel artificial membrane permeability assay for the BBB assay was carried out as previously described with some modifications [16]. Briefly, 20 mg/mL thinned peach extracts in 50% ethanol (*v*/*v*) was diluted to 5 mg/mL with potassium phosphate buffer (10 mmol/L, pH 7.4). Then, 350 μL of 5 mg/mL extracts and 350 μL of buffer were added to each well of the donor plate and the acceptor plate, respectively. The filter membrane of the donor plate was coated with 5 µL of BBB solution. Afterwards, the whole plate was incubated in the dark for 4 h at 37 °C. After incubation, 300 µL of sample from each well in the donor and acceptor plates was taken out, collected and dried. The dried acceptor and donor samples were redissolved in 50 µL of ethanol and analyzed via HPLC-Q-TOF-MS/MS as described below. The permeability across the artificial BBB of each compound was calculated in terms of cm/s, as in [16].

### 2.8. HPLC-Q-TOF-MS/MS Analysis

The dried PLE extracts were dissolved in ACN/water (3:97, *v*/*v*) containing a mixture of internal standards compounds (Table S7, Supplementary Materials) to a final concentration of 3 mg/mL. The samples were vortexed for 30 s and centrifuged at 14,800 rpm for 5 min at 4 °C, and the supernatants were collected and stored at −80 °C until analysis. Aliquots of 5 μL were injected into an HPLC instrument (model 1290) coupled to a Q-TOF mass spectrometer (model 6540 series), both from Agilent Technologies (Waldbronn, Germany). The compounds were separated using a Kinetex PFP column (30 × 2.1 mm, particle size 1.7 µm) equipped with a PFP SecurityGuard™ column, both from Phenomenex (Torrance, CA, USA). The column temperature was held at 45 °C, the flow rate was set to 0.4 mL/min and mobile phases A (H_2_O with 0.1% (*v*/*v*) FA) and B (ACN with 0.1% (*v*/*v*) FA) were used. The gradient was 7~30% B in 4.5 min, 30~100% B in 2 min, 100% B for 1 min, 100~7% B in 0.5 min and 7% B for 2 min with 3 min of post-time. ESI in negative ion mode was used with the following parameters: gas temperature: 325 °C; drying gas: 8 L/min; nebulizer: 35 psig; sheath gas temperature: 350 °C; sheath gas flow: 11 L/min; capillary voltage: 3500 V; nozzle voltage: 1000 V; fragmentor: 120 V; skimmer: 65 V; octapole: 750 V. The *m*/*z* range was from 50 to 1700 for MS and MS/MS. To ensure proper mass accuracy, the spectra were corrected using ions at *m*/*z* 119.0363 (C_5_H_4_N_4_) and 966.0007 (C_18_H_18_O_6_N_3_P_3_F_24_ + formate), which were simultaneously pumped into the ionization source.

The metabolomics data were processed and analyzed using MS-DIAL v4.6 software [20]. The in-house *m*/*z*-retention time library and MS/MS spectra from the NIST20, LipidBLAST and MoNA databases were used for metabolite identification. CUDA, quercetin-d3, reserpine-d9, trans-cinnamic acid-d5 and hippuric acid-d5 internal standards were used for the retention time correction and compound identification using the *m*/*z*-retention time library. Unknown metabolites, duplicated metabolites and isotopes, metabolites with a maximum height below three times the height in the blank samples and metabolites with a maximum height below 1000 units were removed from the list of metabolites. Missing values were imputed by half of the minimum value, and the data were processed by MS-FLO (https://msflo.fiehnlab.ucdavis.edu/#/ accessed on 30 June 2022). The heights of the different adducts ([M-H]^−^, [2M-H]^−^, [M+Cl]^−^ and [M+FA-H]^−^) and fragments from the same compound were combined. The web-based ClassyFire application for conversion (https://cfb.fiehnlab.ucdavis.edu/ accessed on 30 June 2022) was used for compound classification.

All experiments were performed in triplicate, and the results are expressed as means ± standard deviation (SD). Statistical significance (*p* < 0.05) was analyzed using an ANOVA with Tukey’s post hoc test using SPSS 21.0 (SPSS Inc., Chicago, IL, USA). A principal component analysis (PCA), partial least squares discriminant analysis (PLS-DA) and heatmap analyses were performed with MetaboAnalyst 5.0 (https://www.metaboanalyst.ca/home.xhtml accessed on 30 June 2022).

## 3. Results and Discussion

### 3.1. In Vitro Neuroprotection of UAE Extracts

Previous studies have demonstrated an evident divergence in the polyphenol composition, antioxidant activity and immunomodulatory potential of different dried (FD, ND, HAD50, HAD70 and HAD90) thinned peach extracts obtained using UAE [9,10]. In the present study, the in vitro neuroprotective potential of the same extracts was evaluated based on the AChE, BChE and LOX inhibitory activity levels expressed in terms of IC_50_ values (Table 1). Among the different drying methods, the UAE extract obtained after HAD90 treatment exhibited the highest activity levels for AChE (IC_50_ of 231.1 µg/mL), BChE (IC_50_ of 282.6 µg/mL) and LOX (IC_50_ of 93.6 µg/mL) assays (Table 1). This finding suggests that the drying conditions and especially the temperature (IC_50_ values for AChE, BChE and LOX generally decreased as the temperature increased, FD < ND < HAD90 < HAD70 < HAD50) can be important parameters to obtain the bioactive phytochemicals responsible for the neuroprotective potential (as will be discussed in the next sections). In this regard, a recently published study reported that apples treated with convective drying exhibited higher antioxidant activity than apples treated with FD [21].

### 3.2. PLE Optimization

The PLE conditions were optimized using a CCD, as described in Section 2.4, and by selecting the TPC, AChE and LOX inhibitory activity levels and ROS scavenging capacity as response variables. Since previous studies had demonstrated that the extraction yield is not necessarily connected to the neuroprotective potential of different natural extracts [22,23], this response variable was not considered in the PLE optimization model. Moreover, and considering that phenolic compounds can prevent the development of the AD pathology [24,25,26], the TPC was fostered in the optimization process. In this sense, the TPC in the multi-response optimization was given double the value of the other variables. Under these conditions, the final PLE optimal conditions were 48% ethanol and 180 °C (Table S5, Supplementary Materials), similar to the experimental point obtained at 50% ethanol and 180 °C (Table S5, Supplementary Materials). As can be observed in the Pareto charts (Figure S1, Supplementary Materials), both the temperature and solvent composition significantly affect the response variables (TPC, AChE, LOX and ROS). However, it is clear that the temperature contributes more to the optimization model (Figure S2, Supplementary Materials). Table 2 shows that substantial increases in TPC values for all solvent compositions were obtained with the increases in temperature, whereby the highest TPC value (100.1 mg GAE/g) was obtained when the extraction conditions were 50% ethanol at 180 °C (7 times higher than the lowest value obtained with 100% water at 50 °C). This result was expected because an increase in temperature reduces the solvent viscosity, enhancing the mass transfer (and probably the polyphenol content) from the sample to the extraction solvent. Regarding the AChE inhibitory activity, the best value (229.5 µg/mL) was again achieved for 50% ethanol at 180 °C, while the worst value (1868.7 µg/mL) was found in the extract obtained with 100% water at 50 °C. In the case of ROS scavenging and LOX inhibitory capacities, the two best values were obtained with water at 180 °C (IC_50_ of 3.6 µg/mL for ROS and 50.0 µg/mL for LOX) and with 50% ethanol at 180 °C (IC_50_ of 3.9 µg/mL for ROS and 65.4 µg/mL for LOX). However, worse values were obtained for 100% ethanol at the same extraction temperature (IC_50_ of 5.7 µg/mL for ROS and 83.4 µg/mL for LOX), highlighting the importance of the use of more polar solvents to extract bioactive compounds with ROS scavenging and LOX inhibitory capacity.

Finally, an additional experiment was performed at 200 °C to verify the responses of all variables when increasing the extraction temperature (200 °C was the maximum working temperature of the PLE equipment). The results showed that at 200 °C, the PLE extraction yield was similar to that at 180 °C, while the TPC decreased (possibly due to chemical reactions such as degradation processes), and the IC_50_ values for AChE, LOX and ROS were worse. Therefore, the optimum PLE conditions were kept as 50% ethanol and 180 °C.

### 3.3. Extraction Yield, TPC and TFC Analyses of PLE Extracts

Based on the previous optimization step, the polyphenol extraction of all samples was performed using PLE with 50% ethanol at 180 °C, and the extraction yields, TPC values and TFC values were evaluated (Table 3). The lowest extraction yield was obtained for FD (77.6%) and the highest for HAD90 (82.4%), and no significant differences were found between ND, HAD50 and HAD70 samples. The TPC and TFC values of the FD thinned peach extract were the highest (100.7 mg GAE/g for TPC and 15.1 mg QE/g for TFC), followed by the HAD90, HAD70, HAD50 and ND samples. These results indicate that FD is the best method to preserve the phenolic and flavonoid contents in thinned peaches, even though it gave the lowest extraction yield. The previous studies suggest that FD retains more bioactive compounds during the processing of fruit in comparison to other drying methods [27,28]. On the other hand, ND (30 °C) showed the lowest TPC and TFC values, which might be due to the oxidation of polyphenols and flavonoids under the influence of UV radiation and oxygen in the air [29]. In the case of the HAD treatment, significant increases appeared in the TPC and TFC values when the drying temperature was increased from 50 °C to 90 °C. This effect has been previously observed in apricots [30], whereby a higher drying temperature resulted in higher chlorogenic and neochlorogenic acid contents than at lower drying temperatures. This degradation was explained by the influence of the polyphenol oxidase (PPO) enzymatic activity, which might remain active for longer periods during the dehydration process (when the drying temperature is around 55~60 °C), whereas a shorter exposure period is needed to inactivate the enzyme at temperatures of 75~80 °C.

### 3.4. In Vitro Neuroprotection of PLE Extracts

#### 3.4.1. Anti-Cholinergic Activity

In general, the thinned peach extracts exhibited a moderate anti-cholinergic activity compared to galantamine, the reference inhibitor used in this study (see Table 3). The FD thinned peach extract exhibited the highest inhibitory activity levels for AChE and BChE, with IC_50_ values of 238.4 µg/mL and 273.6 µg/mL, while the ND extract showed the lowest activity levels. Regarding the HAD thinned peach extracts, the ChE inhibitory potential increased as the temperature increased. Again, this result was probably correlated with the inhibition of the PPO enzymatic activity as the temperature increased. Moreover, the inhibitory activity levels of AChE and BChE from the different thinned peach extracts were positively correlated with their TPC and TFC values (Table 4), suggesting that the polyphenol content was primarily responsible for the ChE inhibitory activity. In fact, several authors have suggested that the amounts and hydroxyl group positions in the phenolic compound structure are related to the cholinesterase inhibition. Thus, hydroxyl groups act via hydrogen bond formation with specific amino acids in the active sites of cholinesterase enzymes. However, the increase in hydroxyl groups on the side phenyl rings of the phenolic compounds could result in greater AChE inhibition and lower BChE inhibition. This fact would explain the lower IC_50_ values for AChE for the tested extracts [31]. Our data are consistent with the results obtained by Blaszczak et al. (2021), who observed that the highest anti-AChE potential values were correlated with those extracts of kiwi–berry fruits with the highest TPC values [32]. The BChE inhibitory activity was the most affected activity by the PLE extraction method compared to the UAE, being enhanced in most extracts. It is also important to note that the anti-ChE activity of the FD extract dramatically changed after using the PLE system, becoming the most active extract. These results suggest that apart from the drying conditions, the extraction method is an important step that affects the obtention of bioactive molecules.

#### 3.4.2. Anti-Inflammatory Activity

As shown in Table 3, the FD thinned peach extract exhibited the highest LOX inhibitory activity (IC_50_ of 63.0 µg/mL), followed by the HAD90, ND, HAD70 and HAD50 samples. These results were slightly worse than for the positive control (quercetin, IC_50_ of 17.2 µg/mL), so the IC_50_ values of the FD, HAD90 and ND extracts can be considered as moderate (25–100 µg/mL [33]). These results were similar to those obtained from orange juice by-product extracts [16,23], which have shown promising neuroprotective potential. Other studies have also demonstrated the anti-inflammatory properties of different natural products extracts, suggesting that the phenolic compounds are responsible for these effects [33]. The association of the phenolic structure (mainly for flavonoids) and anti-inflammatory activity (as LOX inhibitors) has been extensively discussed, and some structural requirements have been stablished for these associations. The presence of the hydroxyl groups in the flavonoids improved the inhibition capacity of these bioactive molecules due to the flavonoid intercalation between the hydrophobic cavity at the enzyme active site [34,35]. The iron-chelating capacity of the flavonoids has also been proposed as another LOX inhibition mechanism, since LOX possesses ferric iron in its active site and flavonoids could disrupt it [36]. Our results agree well with these publications, as the LOX inhibitory capacity for the different thinned peach extracts were positively correlated to their TPC and TFC values (Table 4). Finally, when comparing the PLE results with those obtained when using UAE, we observed that the LOX inhibitory activity of the FD extract was the most affected, with this extract becoming the most active (IC_50_ of 63.0 µg/mL for PLE vs. 151.9 µg/mL for UAE). The activity levels of the HAD90 and ND extracts were also slightly enhanced, but not for the other extracts. Overall, these results suggest that the performance of the PLE technology is better when using UAE, but it has to be noted that the solvents used for UAE and PLE are different (methanol for UAE and 50% ethanol for PLE), which can also affect the compounds extracted with each technology.

#### 3.4.3. Antioxidant Activity

Generally, the oxidative stress induced by ROS or RNS causes considerable damage to the cell structure and biomolecular functions, resulting in a variety of chronic diseases [37]. Table 3 shows that the highest ROS and RNS scavenging capacity levels were obtained for the FD thinned peach extract (IC_50_ of 3.9 µg/mL for ROS and 42.3% for RNS). In the case of ROS, the IC_50_ value was almost the same as the reference inhibitor (ascorbic acid, IC_50_ of 3.3 µg/mL). Regarding the HAD thinned peach extracts, the ROS and RNS scavenging capacity levels of these extracts increased as the temperature increased (HAD90 > HAD70 > HAD50). On the other hand, the lowest activity was found in the ND extracts. Previous studies have shown that PLE extracts obtained from different natural sources have high antioxidant capacity based on their phenolic composition [22,23]. Phenolic compounds are characterized by possessing phenolic hydroxyl groups that are prone to donate a hydrogen atom or an electron to a free radical, and they have an extended conjugated aromatic system that is used to delocalize an unpaired ROS or RNS electron [38]. In addition, the –CH_2_COOH and –CH=CHCOOH functional groups can promote the antioxidant activity of phenolic acids, which may be related to their ability to donate electrons [39]. As presented in the next section, the abundance levels of several phenolic compounds such as 4-O-caffeoylquinic acid, 4-O-p-coumaroylquinic acid, chlorogenic acid, caffeic acid and isoferulic acid were higher in FD extracts, followed by HAD90, which might explain the higher antioxidant capacity of these extracts.

### 3.5. Characterization of Metabolites in Thinned Peach Extracts from PLE

The HPLC-Q-TOF-MS/MS chemical characterization of the PLE extracts obtained from the dried thinned peaches is presented in Table 5. The results show that 81 compounds belonging to 31 chemical subclasses were tentatively identified. This number was higher than those obtained in our previous works, where a total of 18 compounds were identified in UAE extracts by LC-Q-Orbitrap/MS [9], while 58 compounds were identified by UPLC-ESI-Q-TOF-MS in peaches and nectarines [7]. This enhancement might be a consequence of the use of PLE or the use of a different LC-MS method, as well as the application of the advanced bioinformatic tools and updated databases.

**Figure 1:**
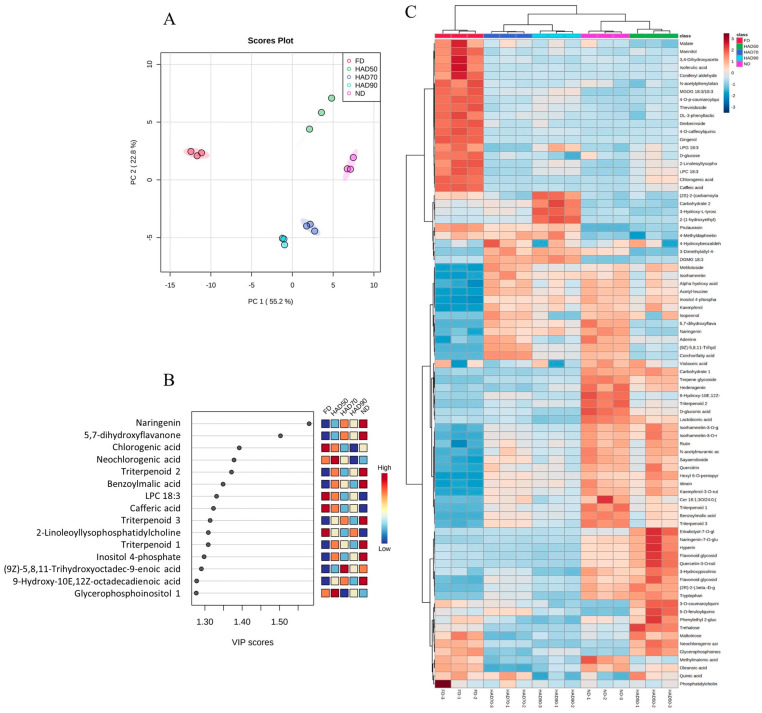
Multivariate statistical analysis showing the projection of metabolites from thinned peach extracts: (**A**) principal component analysis (PCA); (**B**) partial least squares discriminant analysis (PLS-DA); (**C**) HeatMap.

The metabolic compositions of the five PLE extracts were analyzed via PCA, PLS-DA and HeatMap methods (Figure 1). The PCA established two principal components (PC1/PC2) from the metabolites in the different thinned peach extracts, explaining 55.2% (PC1) and 22.8% (PC2) of the variance (Figure 1A). It can be also observed that the FD samples are clearly separated from the other samples, while the HAD90 samples are closer to HAD70 samples and the HAD50 samples are closer to ND samples. The PLS-DA analysis results (Figure 1B) shows a better separation of the samples, and provides 15 metabolites (VIP scores > 1), with naringenin, 5,7-dihydroxyflavanone and chlorogenic acid being the most distinctive variables among the five extracts. Moreover, the HeatMap analysis results (Figure 1C) shows that malate, gingerol, chlorogenic acid, isoferulic acid and 4-O-caffeoylquinic acid are representative metabolites of the FD extract, whereas carbohydrate 2, 3-hydroxy-L-tyrosine, (2S)-2-(carbamoylamino)-4-(methylsulfanyl)butanoic acid and 2-(1-hydroxyethyl)-4-(2-hydroxypropyl)-2H-furan-5-one are representative metabolites of HAD90. Other interesting metabolites commonly present in FD, HAD90 and HAD70 samples are prulaurasin and 4-methyldaphnetin. On the other hand, some glycosides such as naringenin-7-O-glucoside, quercetin-3-O-rutinoside, kaempferol-3-O-rutinoside and isorhamnetin-3-O-glucoside are the characteristic indicators in the HAD50 and ND extracts. These last results are consistent with the literature data, which showed that flavonoids are likely to lose their glycosyl component in the process of heating [40]. The authors of this and other studies have observed that FD is the best method to preserve flavonoids, but they also suggest that because a large percentage of phenolic compounds are bound to the cellular structures, the drying treatments can release these phytochemicals from the matrix to make them more accessible for extraction. In addition, other studies have suggested that the non-glycosylated forms of flavonoids have higher anti-inflammatory capacity than the corresponding glycoside [35], which might explain the lower LOX inhibitory capacity of HAD50 and ND extracts.

### 3.6. Correlation between Metabolites and Neuroprotective Potential

**Figure 2:**
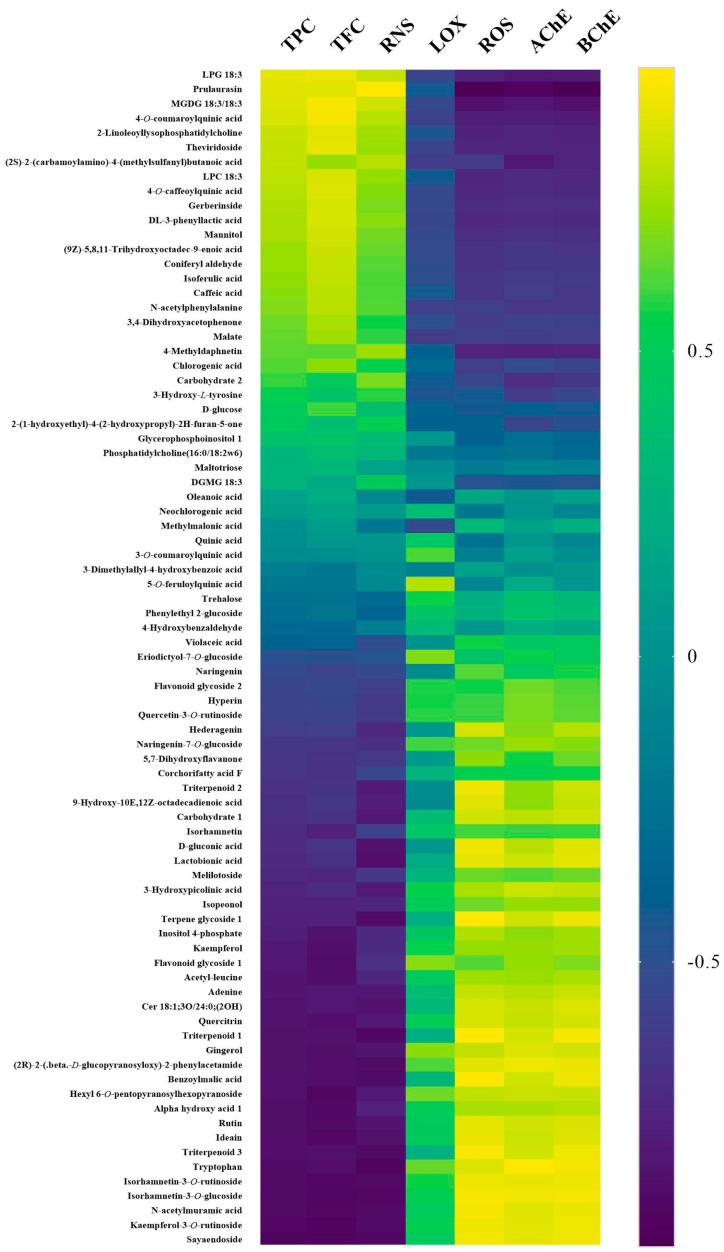
HeatMap of the correlations between metabolites and the neuroprotective potential.

A correlation analysis was then carried out between the 81 tentatively identified metabolites in PLE extracts and the TPC, TFC, RNS, LOX, ROS, AChE and BChE values (Figure 2). Regarding the TPC, TFC and RNS experiments, the yellow grids in the HeatMap indicate that the metabolites are positively correlated to the neuroprotective potential, while the blue grids indicate positive correlations for LOX, ROS, AChE and BchE assays.

The results show that the abundance levels of 20 metabolites (upper part of Figure 2) are positively correlated with the neuroprotective potential, with an average correlation coefficient higher than 0.6. Among these metabolites, 4-O-caffeoylquinic acid, isoferulic acid and caffeic acid are especially relevant because they are commonly present in fruits, tea and coffee [41,42], and they have been reported to possess promising neuroprotective potential [43,44]. Furthermore, Wu et al. (2016) have demonstrated that coniferyl aldehyde can protect neuronal cells from cell death in models of neurodegenerative disorders [45]. Moreover, acetophenone derivatives, including 3,4-dihydroxyacetophenone from the root bark of *Cynanchum wilfordii,* have been proven to possess neuroprotective potential [46]. Another interesting metabolite is prulaurasin (a combination of prunasin and sambunigrin), and prunasin derivatives have also demonstrated neuroprotective potential [47]. Other studies have reported that coumarins, such as 4-methyldaphnetin, have powerful scavenging capacity for superoxide free radicals and peroxides, exerting neuroprotective properties [48]. Finally, several polyphenol glycosides such as kaempferol-3-O-rutinoside, isorhamnetin-3-O-glucoside, isorhamnetin-3-O-rutinoside, naringenin-7-O-glucoside and quercetin-3-O-rutinoside were negatively related to the neuroprotective potential, suggesting that the loss of the glycosyl component during the heating process is beneficial for improving the neuroprotective capacity of the thinned peach extracts.

Overall, the FD thinned peach extract exhibited the best neuroprotective potential, followed by the HAD90, HAD70, HAD50 and ND samples. The abundance levels of 20 metabolites (Table S8, Supplementary Materials) were significantly higher in FD extracts. Among these metabolites, 17 were positively correlated with the neuroprotective potential, such as prulaurasin, chlorogenic acid, 4-O-caffeoylquinic acid and caffeic acid. These two last metabolites are of special interest, as a previous study demonstrated their AChE inhibitory capacity [49]. In the case of the HAD90 thinned peach extract, the abundance levels of 4 metabolites ((2S)-2-(carbamoylamino)-4-(methylsulfanyl)butanoic acid, carbohydrate 2, 2-(1-hydroxyethyl)-4-(2-hydroxypropyl)-2H-furan-5-one and 3-hydroxy-L-tyrosine) were higher, with all of them being positively associated with the neuroprotective potential. This might explain the neuroprotective potential of the HAD90 extract, which was only lower than the FD extract. Finally, the abundance levels of 4, 11 and 16 metabolites were higher in the HAD70, HAD50 and ND extracts, respectively. Many of these metabolites are polyphenols with glycoside linkages, which might result in the decreased activity of these extracts.

### 3.7. BBB Permeability Evaluation

**Figure 3:**
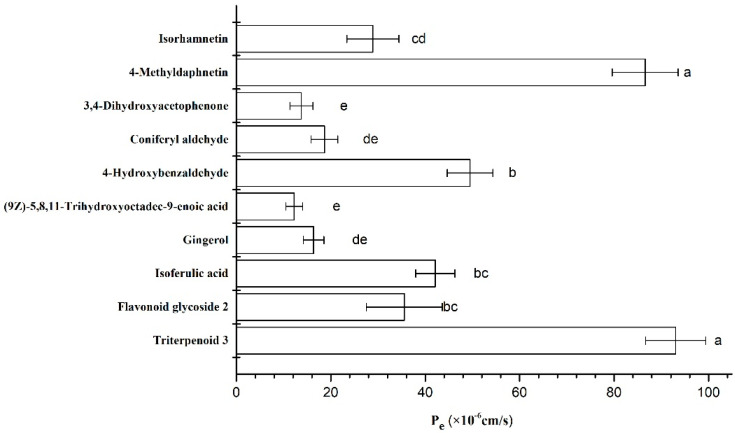
Permeability P_e_ (× 10^−6^ cm/s) of compounds in the thinned peach extracts. Different letters next to the error bars indicate significant differences between compounds after ANOVA with Tukey’s post hoc test, *p*-value < 0.05.

The BBB permeability has been taken as a crucial factor in neuroprotective drug discovery [50] and an artificial BBB method has been optimized and successfully applied to screen compounds from natural products that can penetrate the brain [51]. In the present study, the ability of the identified metabolites found in the five PLE extracts to cross the BBB was evaluated. The results demonstrated that 10 out of 81 metabolites could pass the artificial BBB (Figure 3), and their permeability (P_e_) values ranged from 1.22 × 10^−5^ to 9.30 × 10^−5^ cm/s. These results agree with previous studies that have demonstrated that almost all macromolecular metabolites and above 98% of small molecular weight compounds could not pass the BBB [52]. The metabolite with the highest permeability value was triterpenoid 3, followed by 4-methyldaphnetin, 4-hydroxybenzaldehide and isoferulic acid. Among the metabolites that could cross the BBB, 5 metabolites including 4-methyldaphnetin, isoferulic acid, coniferyl aldehyde, 3,4-dihydroxyacetophenone and (9Z)-5,8,11-Trihydroxyoctadec-9-enoic acid are positively correlated with the neuroprotective potential of the extracts (Figure 2). In addition, the abundance levels of the first four metabolites were higher in the FD thinned peach extract, which together with the above-mentioned results, highlight the promising neuroprotective potential of the FD extract.

## 4. Conclusions

The present study confirms that dried thinned peaches possess neuroprotective potential in view of their in vitro bioactivity and metabolic composition. Compared to UAE, the PLE technology improved the neuroprotective activity of the extracts, with 50:50 ethanol/water at 180 °C being the optimum conditions for the extraction. The TPC, TFC, anti-cholinergic activity, anti-inflammatory activity and antioxidant capacity results demonstrated that the FD thinned peach extract obtained from PLE exhibited the highest neuroprotective activity, followed by the HAD90, HAD70, HAD50 and ND samples. Interestingly, the abundance levels of 20 metabolites in the FD extract were significantly higher than other extracts, and 17 of them were positively correlated to the neuroprotective potential. Furthermore, most of the metabolites that could pass the BBB were more abundant in the FD extract.

## Figures and Tables

**Table 1:** AChE, BChE and LOX inhibitory activity levels (IC_50_ μg/mL) of thinned peach extracts obtained using UAE.

Samples	AChE	BChE	LOX
FD	547.3 ± 40 ^a^	894.1 ± 43 ^a^	151.9 ± 19 ^a^
ND	391.1 ± 36 ^bc^	942.5 ± 99 ^a^	131.2 ± 15 ^ab^
HAD50	425.6 ± 43 ^b^	844.6 ± 55 ^a^	104.2 ± 6 ^bc^
HAD70	359.5 ± 19 ^c^	567.3 ± 7 ^b^	163.1 ± 12 ^a^
HAD90	231.1 ± 20 ^d^	282.6 ± 20 ^c^	93.6 ± 10 ^c^

Different letters in the same column indicate significant differences between samples after the ANOVA with Tukey’s post hoc test, *p*-value < 0.05.

**Table 2:** Results observed in the response variables during the optimization of PLE conditions for thinned peach extraction.

No. (Unit)	Temp. (°C)	Solvent Composition	Yield (%)	TPC (mg GAE/g)	AChE (IC_50_ µg/mL)	LOX (IC_50_ µg/mL)	ROS (IC_50_ µg/mL)
1	115	Water	38.5	22.9 ± 2	1634.8 ± 120	237.4 ± 15	12.4 ± 0.9
2	50	Water	31.4	13.8 ± 0.6	1868.7 ± 129	288.3 ± 33	16.7 ± 0.1
3	115	50% ethanol	46.9	37.4 ± 3	1255.4 ± 75	385.3 ± 29	9.2 ± 0.4
4	115	Ethanol	48.5	37.6 ± 2	1259.1 ± 93	546.5 ± 49	9.2 ± 0.5
5	115	50% ethanol	43.7	36.1 ± 1	1174.0 ± 114	393.0 ± 31	9.6 ± 0.9
6	50	Ethanol	34.2	29.1 ± 0.5	1377.9 ± 130	717.3 ± 68	12.1 ± 0.8
7	50	50% ethanol	38.4	26.0 ± 2	1340.0 ± 58	476.5 ± 34	11.7 ± 1
8 *	180	50% ethanol	79.3	100.1 ± 5	229.5 ± 22	65.4 ± 5	3.9 ± 0.2
9	115	50% ethanol	42.0	38.3 ± 0.9	1205.5 ± 120	377.4 ± 41	9.3 ± 0.9
10	180	Ethanol	60.1	81.4 ± 3	347.3 ± 35	83.4 ± 6	5.7 ± 0.7
11	180	Water	77.0	82.3 ± 3	384.8 ± 32	50.0 ± 5	3.6 ± 0.4
12	115	50% ethanol	41.2	40.7 ± 0.5	1196.5 ± 113	325.1 ± 16	9.5 ± 0.4
13 ^#^	200	50% ethanol	80.0	91.3 ± 2	285.5 ± 12	72.9 ± 6	4.2 ± 0.3

* Optimal point. ^#^ Not included in the optimization model.

**Table 3:** Yield, TPC, TFC and neuroprotective potential evaluation of thinned peach extracts from PLE under optimized conditions.

Samples	Yield (%)	TPC (mg GAE/g)	TFC (mg QE/g)	AChE (IC_50_ µg/mL)	BChE (IC_50_ µg/mL)	LOX (IC_50_ µg/mL)	ROS (IC_50_ µg/mL)	RNS (%)
FD	77.6 ± 0.3 ^b^	100.7 ± 2 ^a^	15.1 ± 0.5 ^a^	238.4 ± 8 ^e^	273.6 ± 4 ^e^	63.0 ± 3 ^e^	3.9 ± 0.2 ^e^	42.3 ± 1 ^b^
ND	80.3 ± 2 ^ab^	58.5 ± 1 ^d^	5.2 ± 0.1 ^d^	447.3 ± 5 ^a^	553.3 ± 7 ^a^	96.0 ± 4 ^c^	7.5 ± 0.3 ^a^	15.2 ± 0.2 ^e^
HAD50	78.8 ± 2 ^ab^	63.2 ± 2 ^cd^	5.6 ± 0.2 ^cd^	426.9 ± 4 ^b^	482.6 ± 2 ^b^	221.4 ± 4 ^a^	6.2 ± 0.1 ^b^	21.5 ± 0.9 ^d^
HAD70	80.5 ± 1 ^ab^	65.1 ± 1 ^c^	6.4 ± 0.3 ^c^	377.4 ± 7 ^c^	424.5 ± 5 ^c^	186.8 ± 2 ^b^	5.4 ± 0.2 ^c^	26.6 ± 2 ^c^
HAD90	82.4 ± 0.2 ^a^	91.3 ± 3 ^b^	11.2 ± 0.4 ^b^	260.3 ± 6 ^d^	318.9 ± 5 ^d^	81.3 ± 7 ^d^	4.8 ± 0.3 ^d^	38.9 ± 3 ^b^
Galantamine				1.2 ± 0.1 ^f^	16.1 ± 1 ^f^			
Quercetin						17.2 ± 2 ^f^		
Ascorbic acid							3.3 ± 0.6 ^e^	83.8 ± 4 ^a^

Different letters in the same column indicate significant differences between samples after ANOVA with Tukey’s post hoc test, *p*-value < 0.05. The RNS values are expressed as the percentage of inhibition (%) of nitrogen species with respect to the control at the maximum concentration tested (0.8 mg/mL for extracts and ascorbic acid).

**Table 4:** Correlations among the TPC, TFC and neuroprotective potential values of PLE thinned peach extracts.

	TPC	TFC	ACHE	BCHE	LOX	ROS	RNS
TPC	1	0.99 **	−0.98 **	−0.95 **	−0.67 **	−0.89 **	0.95 **
TFC	0.99 **	1	−0.96 **	−0.93 **	−0.70 **	−0.88 **	0.93 **

** significant correlation at the 0.01 level (*p* < 0.01).

**Table 5:** Tentatively identified metabolites in all thinned peach PLE extracts using LC-Q-TOF-MS/MS.

Retention Time (min)	Metabolite Name	Adduct Type or Fragment	*m/z*	Formula	Chemical Subclass
0.811	3-*O*-coumaroylquinic acid	[M-H]^−^	337.0791	C_16_H_18_O_8_	Alcohols and polyols
1.780	4-*O*-caffeoylquinic acid	[M-H-H_2_O]^−^/ [M-H-C_7_H_10_O_5_]^−^	335.0780/ 161.0244	C_16_H_18_O_9_	Alcohols and polyols
2.258	4-*O*-*p*-coumaroylquinic acid	[M-H-H_2_O]^−^	319.0828	C_16_H_18_O_7_	Alcohols and polyols
1.072	5-*O*-feruloylquinic acid	[M-H]^−^	367.1035	C_17_H_20_O_9_	Alcohols and polyols
1.018	Chlorogenic acid	[M-H]^−^/ [M-H-C_9_H_6_O_3_]^−^/ [2M-H]^−^	353.0888/ 191.0564/ 707.1846	C_16_H_18_O_9_	Alcohols and polyols
0.238	Inositol 4-phosphate	[M-H]^−^	259.0246	C_6_H_13_O_9_P	Alcohols and polyols
0.572	Neochlorogenic acid	[2M-H]^−^/ [M-H-C_7_H_10_O_5_]^−^/ [M-H-C_8_H_10_O_7_]^−^/ [M-H]^−^	707.1853/ 179.0353/ 135.0446/ 353.0890	C_16_H_18_O_9_	Alcohols and polyols
0.259	Quinic acid	[M-H]^−^/ [2M-H]^−^	191.0590/ 383.1260	C_7_H_12_O_6_	Alcohols and polyols
1.214	*Alpha hydroxy acid 1* ((4E)-8-hydroxy-4-(1-hydroxypropan-2-ylidene)-10-oxatricyclo[7.2.1.0]dodecane-8-carboxylic acid)	[M-H]^−^	281.1395	C_15_H_22_O_5_	Alpha hydroxy acids and derivatives
0.487	(2S)-2-(carbamoylamino)-4-(methylsulfanyl)butanoic acid	[2M-H]^−^	383.1107	C_6_H_12_N_2_O_3_S	Amino acids, peptides, and analogues
0.415	3-Hydroxy-*L*-tyrosine	[M-H]^−^	196.0623	C_9_H_11_NO_4_	Amino acids, peptides, and analogues
0.996	Acetyl-leucine	[M-H]^−^	172.0980	C_8_H_15_NO_3_	Amino acids, peptides, and analogues
1.575	N-acetylphenylalanine	[M-H]^−^	206.0825	C_11_H_13_NO_3_	Amino acids, peptides, and analogues
4.260	3-Dimethylallyl-4-hydroxybenzoic acid	[M-H]^−^	205.0867	C_12_H_14_O_3_	Benzoic acids and derivatives
1.851	Benzoylmalic acid	[M-H-C_4_H_4_O_4_]^−^/ [M-H]^−^	121.0293/ 237.0405	C_11_H_10_O_6_	Benzoic acids and derivatives
0.279	Malate	[M-H]^−^	133.0157	C_4_H_6_O_5_	Beta hydroxy acids and derivatives
0.631	*Carbohydrate 1* ((2R,3S,4S,5R,6R)-5-[(2S,3R,4R)-3,4-dihydroxy-4-(hydroxymethyl)oxolan-2-yl]oxy-2-(hydroxymethyl)-6-[2-(4-hydroxyphenyl)ethoxy]oxane-3,4-diol)	[M+FA-H]^−^	477.1605	C_19_H_28_O_11_	Carbohydrates and carbohydrate conjugates
0.328	*Carbohydrate 2* (3-[(2S,3R,4S,5S,6R)-6-[[(2R,3R,4R)-3,4-dihydroxy-4-(hydroxymethyl)oxolan-2-yl]oxymethyl]-3,4,5-trihydroxyoxan-2-yl]oxy-2-methylpyran-4-one)	[M+FA-H]^−^	465.1282	C_17_H_24_O_12_	Carbohydrates and carbohydrate conjugates
0.252	D-gluconic acid	[M-H]^−^	195.0531	C_6_H_12_O_7_	Carbohydrates and carbohydrate conjugates
0.262	D-glucose	[M-H]^−^	179.0583	C_6_H_12_O_6_	Carbohydrates and carbohydrate conjugates
0.240	Maltotriose	[M+FA-H]^−^/ [M+Cl]^−^	549.1705/ 539.1381	C_18_H_32_O_16_	Carbohydrates and carbohydrate conjugates
0.243	Mannitol	[M+Cl]^−^/ [M-H]^−^/ [M+FA-H]^−^	217.0499/ 181.0737/ 227.0794	C_6_H_14_O_6_	Carbohydrates and carbohydrate conjugates
1.146	Melilotoside	[M+FA-H]^−^	371.0993	C_15_H_18_O_8_	Carbohydrates and carbohydrate conjugates
0.311	N-acetylmuramic acid	[M-H]^−^	292.1045	C_11_H_19_NO_8_	Carbohydrates and carbohydrate conjugates
1.308	Phenylethyl 2-glucoside	[M+FA-H]^−^	329.1236	C_14_H_20_O_6_	Carbohydrates and carbohydrate conjugates
1.137	Prulaurasin	[M+FA-H]^−^/ [M+Cl]^−^	340.1050/ 330.0758	C_14_H_17_NO_6_	Carbohydrates and carbohydrate conjugates
1.421	Sayaendoside	[M+FA-H]^−^/ [M-H]^−^	461.1720/ 415.1613	C_19_H_28_O_10_	Carbohydrates and carbohydrate conjugates
0.239	Trehalose	[M+Cl]^−^/ [M+FA-H]^−^/ [M-H]^−^	377.0876/ 387.1167/ 341.1112	C_12_H_22_O_11_	Carbohydrates and carbohydrate conjugates
1.012	3,4-Dihydroxyacetophenone	[M-H]^−^	151.0400	C_8_H_8_O_3_	Carbonyl compounds
1.220	4-Hydroxybenzaldehyde	[M-H]^−^	121.0288	C_7_H_6_O_2_	Carbonyl compounds
2.175	Isopeonol	[M-H]^−^	165.0414	C_9_H_10_O_3_	Carbonyl compounds
7.074	Cer 18:1;3O/24:0;(2OH)	[M-H]^−^	680.6199	C_42_H_83_NO_5_	Ceramides
1.401	Gerberinside	[M-H]^−^	337.0793	C_16_H_18_O_8_	Coumarin glycosides
0.342	Methylmalonic acid	[M-H]^−^	117.0210	C_4_H_6_O_4_	Dicarboxylic acids and derivatives
0.802	Violaceic acid	[M-H-C_8_H_6_O_3_]^−^	137.0099	C_15_H_12_O_6_	Diphenylethers
4.629	(9Z)-5,8,11-Trihydroxyoctadec-9-enoic acid	[M-H]^−^	329.2348	C_18_H_34_O_5_	Fatty acids and conjugates
0.401	(2R)-2-(.βeta.-*D*-glucopyranosyloxy)-2-phenylacetamide	[M+CHO_2_]^−^	358.1162	C_14_H_19_NO_7_	Fatty acyl glycosides
1.900	Hexyl 6-*O*-pentopyranosylhexopyranoside	[M+CHO_2_]^−^	441.1979	C_17_H_32_O_10_	Fatty acyl glycosides
0.247	Lactobionic acid	[M-H]^−^	357.1067	C_12_H_22_O_12_	Fatty acyl glycosides
5.512	5,7-dihydroxyflavanone	[M-H]^−^	255.0665	C_15_H_12_O_4_	Flavans
4.379	Naringenin	[M-H]^−^	271.0619	C_15_H_12_O_5_	Flavans
4.838	Isorhamnetin	[M-H]^−^	315.0510	C_16_H_12_O_7_	Flavones
4.659	Kaempferol	[M-H]^−^	285.0413	C_15_H_10_O_6_	Flavones
4.016	*Flavonoid glycoside 1* ([6-[2-(3,4-dihydroxyphenyl)-8-hydroxy-4-oxochromen-7-yl]oxy-3,4,5-trihydroxyoxan-2-yl]methyl (E)-3-(4-hydroxyphenyl)prop-2-enoate)	[M-H]^−^	593.1296	C_30_H_26_O_13_	Flavonoid glycosides
1.826	*Flavonoid glycoside 2* (2-(3,4-Dihydroxyphenyl)-5,7-dihydroxy-4-oxo-4H-chromen-3-yl 4-*O*-hexopyranosylhexopyranoside)	[M-H]^−^	625.1416	C_27_H_30_O_17_	Flavonoid glycosides
2.334	Eriodictyol-7-*O*-glucoside	[M-H]^−^	449.1093	C_21_H_22_O_11_	Flavonoid glycosides
2.371	Hyperin	[M-H]^−^	463.0891	C_21_H_20_O_12_	Flavonoid glycosides
2.723	Ideain	[M-2H]^−^	447.0947	C_21_H_21_ClO_11_	Flavonoid glycosides
2.862	Isorhamnetin-3-*O*-glucoside	[M-H]^−^	477.1045	C_22_H_22_O_12_	Flavonoid glycosides
2.768	Isorhamnetin-3-*O*-rutinoside	[M-H]^−^	623.1625	C_28_H_32_O_16_	Flavonoid glycosides
2.632	Kaempferol-3-*O*-rutinoside	[M-H]^−^	593.1521	C_27_H_30_O_15_	Flavonoid glycosides
2.786	Naringenin-7-*O*-glucoside	[M-H-C_6_H_10_O_5_]^−^/ [M-H]^−^/ [M+Cl]^−^	271.0606/ 433.1142/ 469.0901	C_21_H_22_O_10_	Flavonoid glycosides
2.302	Quercetin-3-*O*-rutinoside	[M-H]^−^	609.1469	C_27_H_30_O_16_	Flavonoid glycosides
2.575	Quercitrin	[M-H]^−^	447.0937	C_21_H_20_O_11_	Flavonoid glycosides
2.107	Rutin	[M-H]^−^	609.1474	C_27_H_30_O_16_	Flavonoid glycosides
0.480	2-(1-hydroxyethyl)-4-(2-hydroxypropyl)-2H-furan-5-one	[M+Cl]^−^	221.0566	C_9_H_14_O_4_	Furanones
6.049	2-Linoleoyllysophosphatidylcholine	[M+FA-H]^−^	564.3302	C_26_H_50_NO_7_P	Glycerophosphocholines
5.948	LPC 18:3	[M+FA-H]^−^	562.3145	C_26_H_48_NO_7_P	Glycerophosphocholines
6.802	Phosphatidylcholine(16:0/18:2w6)	[M+CHO_2_]^−^	802.5610	C_42_H_80_NO_8_P	Glycerophosphocholines
5.989	LPG 18:3	[M-H]^−^	505.2558	C_24_H_43_O_9_P	Glycerophosphoglycerols
5.643	*Glycerophosphoinositol 1* (D-myo-Inositol, 1-[2-hydroxy-3-[(1-oxo-9,12-octadecadienyl)oxy]propyl hydrogen phosphate], [S-(Z,Z)]-)	[M-H]^−^	595.2888	C_27_H_49_O_12_P	Glycerophosphoinositols
5.783	DGMG 18:3	[M+FA-H]^−^	721.3654	C_33_H_56_O_14_	Glycosylglycerols
6.669	MGDG 18:3/18:3	[M+HCOO]^−^	819.5268	C_45_H_74_O_10_	Glycosylglycerols
0.981	Caffeic acid	[M-H]^−^	179.0349	C_9_H_8_O_4_	Hydroxycinnamic acids and derivatives
1.857	Isoferulic acid	[M-H]^−^	193.0504	C_10_H_10_O_4_	Hydroxycinnamic acids and derivatives
1.724	4-Methyldaphnetin	[M-H]^−^	191.0340	C_10_H_8_O_4_	Hydroxycoumarins
1.105	Tryptophan	[M-H]^−^	203.0827	C_11_H_12_N_2_O_2_	Indolyl carboxylic acids and derivatives
5.976	9-Hydroxy-10E,12Z-octadecadienoic acid	[M-H]^−^	295.2278	C_18_H_32_O_3_	Lineolic acids and derivatives
4.228	Corchorifatty acid F	[M-H]^−^/ [M+Cl]^−^	327.2181/ 363.1942	C_18_H_32_O_5_	Lineolic acids and derivatives
1.576	Coniferyl aldehyde	[M-H]^−^	177.0551	C_10_H_10_O_3_	Methoxyphenols
5.460	Gingerol	[M-H]^−^	293.1810	C_17_H_26_O_4_	Methoxyphenols
1.377	DL-3-phenyllactic acid	[M-H]^−^	165.0555	C_9_H_10_O_3_	Phenylpropanoic acids
0.334	Adenine	[M-H]^−^	134.0488	C_5_H_5_N_5_	Purines and purine derivatives
0.390	3-Hydroxypicolinic acid	[M-H]^−^	138.0202	C_6_H_5_NO_3_	Pyridinecarboxylic acids and derivatives
1.443	*Terpene glycoside 1* ((2R,3R,4S,5S,6R)-2-[6-hydroxy-3-[(E)-3-hydroxybut-1-enyl]-2,4,4-trimethylcyclohexyl]oxy-6-(hydroxymethyl)oxane-3,4,5-triol)	[M+Cl]^−^	425.2031	C_19_H_34_O_8_	Terpene glycosides
0.453	Theviridoside	[M+FA-H]^−^	449.1303	C_17_H_24_O_11_	Terpene glycosides
5.685	*Triterpenoid 1* ((1R,2R,4aS,6aS,6bR,10S,12aR,14bS)-1,8,10-trihydroxy-1,2,6a,6b,9,9,12a-heptamethyl-2,3,4,5,6,6a,7,8,8a,10,11,12,13,14b-tetradecahydropicene-4a-carboxylic acid)	[M-H]^−^	487.3437	C_30_H_48_O_5_	Triterpenoids
5.803	*Triterpenoid 2* ((1S,4aR,6aS,6bR,10R,11R,12aR,14bS)-1,10,11-trihydroxy-2,2,6a,6b,9,9,12a-heptamethyl-1,3,4,5,6,6a,7,8,8a,10,11,12,13,14b-tetradecahydropicene-4a-carboxylic acid)	[M-H]^−^	487.3432	C_30_H_48_O_5_	Triterpenoids
5.552	*Triterpenoid 3* ((1S,4aR,6aS,6bR,9S,10R,11R,12aR,14bS)-1,10,11-trihydroxy-9-(hydroxymethyl)-2,2,6a,6b,9,12a-hexamethyl-1,3,4,5,6,6a,7,8,8a,10,11,12,13,14b-tetradecahydropicene-4a-carboxylic acid)	[M-H]^−^	503.3377	C_30_H_48_O_6_	Triterpenoids
5.980	Hederagenin	[M-H]^−^	471.3482	C_30_H_48_O_4_	Triterpenoids
6.225	Oleanoic acid	[M-H]^−^	455.3534	C_30_H_48_O_3_	Triterpenoids

## Data Availability

Data is contained within the article or Supplementary Materials.

## Supplementary Materials

The following supporting information can be downloaded at: https://www.mdpi.com/article/10.3390/foods11162464/s1. Table S1: Analysis of variance for TPC for response surface modeling showing linear, quadratic and interaction relations and coefficients for model prediction. Table S2: Analysis of variance for AChE for response surface modeling showing linear, quadratic and interaction relations and coefficients for model prediction. Table S3: Analysis of variance for LOX for response surface modeling showing linear, quadratic and interaction relations and coefficients for model prediction. Table S4: Analysis of variance for ROS for response surface modeling showing linear, quadratic and interaction relations and coefficients for model prediction. Table S5: PLE conditions, desirability, predicted response values at the optimum predicted by the model and experimental response values for the selected optimum (50% ethanol at 180 °C). Table S6: Multiple response optimization. Table S7: MS1 (*m/z*) and RT (min) of the internal standards used. Table S8: Abundance levels of tentatively identified metabolites in thinned peach PLE extracts. Figure S1: Response surfaces for each response variable and their corresponding standardized Pareto charts: A, TPC (mg GAE/mL); B, IC50 AChE (µg/mL); C, IC50 LOX (µg/mL); D, IC50 ROS (µg/mL). Figure S2: Desirability response surface used to optimize all response variables.
