# Supplementary material for: Neuroprotective Potential of Thinned Peaches Extracts Obtained by Pressurized Liquid Extraction after Different Drying Processes
Source: Foods. 2022 Aug 16;11(16):2464. doi: 10.3390/foods11162464 (PMC9407205; doi:10.3390/foods11162464)
Supplement: Supplementary file 1 [file foods-11-02464-s001.zip › Figure S1.pdf]

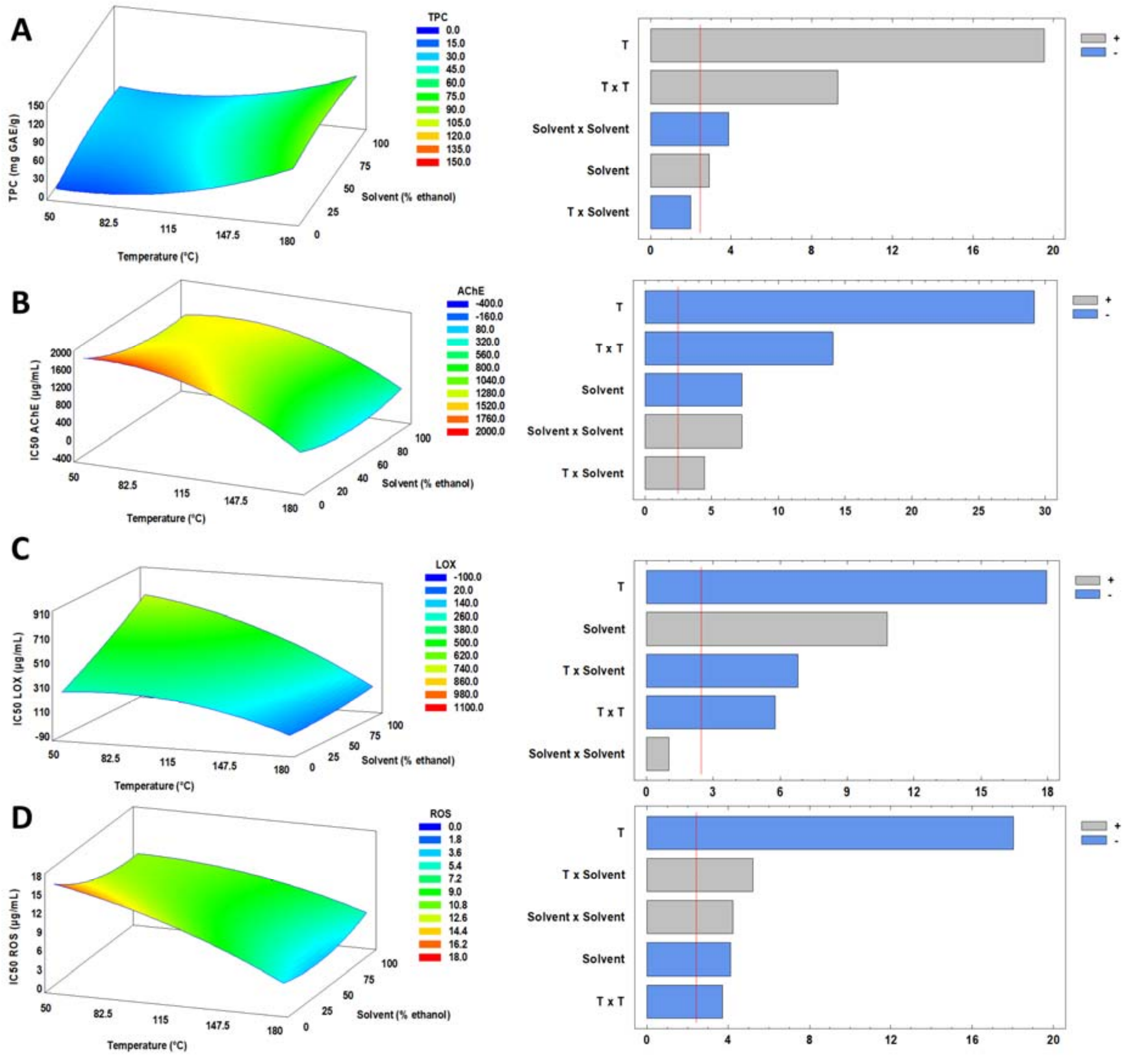

Figure S1. Response surfaces for each response variable and their corresponding Standardized Pareto charts: **A**, TPC (mg GAE/mL); **B**, IC_{50} AChE (µg/mL); **C**, IC_{50} LOX (µg/mL); **D**, IC_{50} ROS (µg/mL).
