# Supplementary material for: Neuroprotective Potential of Thinned Peaches Extracts Obtained by Pressurized Liquid Extraction after Different Drying Processes
Source: Foods. 2022 Aug 16;11(16):2464. doi: 10.3390/foods11162464 (PMC9407205; doi:10.3390/foods11162464)
Supplement: Supplementary file 1 [file foods-11-02464-s001.zip › Table S1.pdf]

Table S1. Analysis of Variance for TPC for response surface modeling showing linear, quadratic and interaction relations and coefficient for model prediction.

Source	*Sum of Squares*	*Df*	*CE*		*F-Ratio*	*P-Value*
Model			β_0	32.0586		
Temperature (°C)	6335.55	1	β_1	-0.699	1662.02	0.0001*
Solvent composition (% ethanol)	140.554	1	β_2	0.627	36.87	0.0090*
Temperature^2	1430.98	1	$\beta_{1,1}$	5.48×10^{-3}	375.39	0.0003*
Temperature x Solvent composition	66.0156	1	$\beta_{1,2}$	-1.25×10^{-3}	17.32	0.0252*
Solvent composition^2	248.842	1	$\beta_{2,2}$	-3.86×10^{-3}	65.28	0.0040*
Lack-of-fit	87.8714	3			7.68	0.0640
Pure error	11.4359	3				
Total (corr.)	8083.68	11				

Df (degree of freedom)

CE (coefficients of regression equation)

* Denotes statistical differences ($p < 0.05$)

R-squared = 98.7715 percent

R-squared (adjusted for d.f.) = 97.7478 percent

Standard Error of Est. = 1.95242

Mean absolute error = 2.44611

Durbin-Watson statistic = 2.76781 (P=0.9638)

Lag 1 residual autocorrelation = -0.412639
