# Supplementary material for: Neuroprotective Potential of Thinned Peaches Extracts Obtained by Pressurized Liquid Extraction after Different Drying Processes
Source: Foods. 2022 Aug 16;11(16):2464. doi: 10.3390/foods11162464 (PMC9407205; doi:10.3390/foods11162464)
Supplement: Supplementary file 1 [file foods-11-02464-s001.zip › Table S2.pdf]

Table S2. Analysis of Variance for AChE for response surface modeling showing linear, quadratic and interaction relations and coefficient for model prediction.

Source	*Sum of Squares*	*Df*	*CE*		*F-Ratio*	*P-Value*
Model			β_0	1487.18		
Temperature (°C)	2.19×10^6	1	β_1	12.7899	1857.19	0.0001*
Solvent composition (% ethanol)	136179	1	β_2	-16.0048	115.48	0.0017*
Temperature^2	510918	1	$\beta_{1,1}$	-0.1036	433.27	0.0002*
Temperature x Solvent composition	51379.3	1	$\beta_{1,2}$	0.0348	43.57	0.0071*
Solvent composition^2	134443	1	$\beta_{2,2}$	0.0898	114.01	0.0018*
Lack-of-fit	11902.9	3			3.36	0.1728
Pure error	3537.660	3				
Total (corr.)	2.92×10^6	11				

Df (degree of freedom)

CE (coefficients of regression equation)

* Denotes statistical differences ($p < 0.05$)

R-squared = 99.4717 percent

R-squared (adjusted for d.f.) = 99.0314 percent

Standard Error of Est. = 34.3398

Mean absolute error = 31.2453

Durbin-Watson statistic = 2.40353 (P=0.8141)

Lag 1 residual autocorrelation = -0.281655
