# Supplementary material for: Neuroprotective Potential of Thinned Peaches Extracts Obtained by Pressurized Liquid Extraction after Different Drying Processes
Source: Foods. 2022 Aug 16;11(16):2464. doi: 10.3390/foods11162464 (PMC9407205; doi:10.3390/foods11162464)
Supplement: Supplementary file 1 [file foods-11-02464-s001.zip › Table S3.pdf]

Table S3. Analysis of Variance for LOX for response surface modeling showing linear, quadratic and interaction relations and coefficient for model prediction.

Source	*Sum of Squares*	*Df*	*CE*		*F-Ratio*	*P-Value*
Model			β_0	141.015		
Temperature (°C)	274494	1	β_1	3.85083	291.08	0.0004*
Solvent composition (% ethanol)	99171.2	1	β_2	5.26086	105.17	0.0020*
Temperature^2	28418.5	1	$\beta_{1,1}$	-0.0244	30.14	0.0119*
Temperature x Solvent composition	39132.8	1	$\beta_{1,2}$	-0.0304	41.50	0.0076*
Solvent composition^2	840.877	1	$\beta_{2,2}$	0.0071	0.89	0.4147
Lack-of-fit	2271.84	3			0.80	0.5694
Pure error	2829.01	3				
Total (corr.)	447149	11				

Df (degree of freedom)

CE (coefficients of regression equation)

* Denotes statistical differences ($p < 0.05$)

R-squared = 98.8593 percent

R-squared (adjusted for d.f.) = 97.9086 percent

Standard Error of Est. = 30.7083

Mean absolute error = 17.3321

Durbin-Watson statistic = 1.43101 (P=0.0817)

Lag 1 residual autocorrelation = -0.0202073
