# Supplementary material for: Neuroprotective Potential of Thinned Peaches Extracts Obtained by Pressurized Liquid Extraction after Different Drying Processes
Source: Foods. 2022 Aug 16;11(16):2464. doi: 10.3390/foods11162464 (PMC9407205; doi:10.3390/foods11162464)
Supplement: Supplementary file 1 [file foods-11-02464-s001.zip › Table S4.pdf]

Table S4. Analysis of Variance for ROS for response surface modeling showing linear, quadratic and interaction relations and coefficient for model prediction.

Source	*Sum of Squares*	*Df*	*CE*		*F-Ratio*	*P-Value*
Model			β_0	18.3692		
Temperature (°C)	120.871	1	β_1	-0.01798	3637.04	0.0001*
Solvent composition (% ethanol)	6.32427	1	β_2	-0.13989	190.30	0.0008*
Temperature^2	5.13375	1	$\beta_{1,1}$	-3.28×10^{-4}	154.48	0.0011*
Temperature x Solvent composition	10.1124	1	$\beta_{1,2}$	4.89×10^{-4}	304.28	0.0004*
Solvent composition^2	6.63602	1	$\beta_{2,2}$	6.31×10^{-4}	199.68	0.0008*
Lack-of-fit	2.13108				21.37	0.0158*
Pure error	0.0997	6				
Total (corr.)	148.402	11				

Df (degree of freedom)

CE (coefficients of regression equation)

* Denotes statistical differences ($p < 0.05$)

R-squared = 98.4968 percent

R-squared (adjusted for d.f.) = 97.2441 percent

Standard Error of Est. = 0.18232

Mean absolute error = 0.351806

Durbin-Watson statistic = 2.65649 (P=0.9347)

Lag 1 residual autocorrelation = -0.378735
