# Supplementary material for: Neuroprotective Potential of Thinned Peaches Extracts Obtained by Pressurized Liquid Extraction after Different Drying Processes
Source: Foods. 2022 Aug 16;11(16):2464. doi: 10.3390/foods11162464 (PMC9407205; doi:10.3390/foods11162464)
Supplement: Supplementary file 1 [file foods-11-02464-s001.zip › Table S5.pdf]

Table S5. PLE conditions, desirability, predicted response values at the optimum predicted by the model and experimental response values for the selected optimum (50% ethanol at 180 ºC).

	Predicted Optimum	*Experimental Optimum (50% ethanol at 180 ºC)*
Temperature (ºC)	180.0	180
Solvent composition (% ethanol)	47.62	50
**Desirability**	0.965072	0.964926
**Response**		
TPC	94.3	100.1±4.6
AChE	173.1	229.5±21.7
LOX	53.0	65.4±5.4
ROS	3.5	3.9±0.2
