# Supplementary material for: Neuroprotective Potential of Thinned Peaches Extracts Obtained by Pressurized Liquid Extraction after Different Drying Processes
Source: Foods. 2022 Aug 16;11(16):2464. doi: 10.3390/foods11162464 (PMC9407205; doi:10.3390/foods11162464)
Supplement: Supplementary file 1 [file foods-11-02464-s001.zip › Table S6.pdf]

Table S6. Multiple response optimization.

Thinned Peach Extract							*Predicted*	*Observed*
**No. (Unit)**	**Temp (ºC)**	**Solvent Composition**	*TPC*	*AChE*	*LOX*	*ROS*	*Desirability*	*Desirability*
5	115	Water	22.9	1634.8	237.4	12.4	0.159286	0.140521
4	50	Water	13.8	1868.7	288.3	16.7	0.0	0.0
8	115	50% ethanol	37.4	1255.4	385.3	9.2	0.316335	0.300841
2	115	Ethanol	37.6	1259.1	546.5	9.2	0.235739	0.255469
9	115	50% ethanol	36.1	1174.0	393.0	9.6	0.316335	0.295834
1	50	Ethanol	29.1	1377.9	717.3	12.1	0.0	0.0
7	50	50% ethanol	26.0	1340.0	476.5	11.7	0.184334	0.174231
12	180	50% ethanol	100.1	229.5	65.4	3.9	0.964926	0.994202
10	115	50% ethanol	38.3	1205.5	377.4	9.3	0.316335	0.314133
3	180	Ethanol	81.4	347.3	83.4	5.7	0.845313	0.827505
6	180	Water	82.3	384.8	50.0	3.6	0.863034	0.869225
11	115	50% ethanol	40.7	1196.5	325.1	9.5	0.316335	0.339606

This procedure helps determining the combination of experimental factors which simultaneously optimize several responses. The output also shows the desirability function evaluated at each point in the design. Among the experimental points in the design, maximum desirability is achieved at run 12 (50% ethanol at 180 ºC).
