# Supplementary material for: Neuroprotective Potential of Thinned Peaches Extracts Obtained by Pressurized Liquid Extraction after Different Drying Processes
Source: Foods. 2022 Aug 16;11(16):2464. doi: 10.3390/foods11162464 (PMC9407205; doi:10.3390/foods11162464)
Supplement: Supplementary file 1 [file foods-11-02464-s001.zip › Table S7.pdf]

Table S7. MS1 (*m/z*) and RT (min) of the internal standards used.

**Internal standard**	**Concentration (μg/mL)**	**MS1 (*m/z*)**	**Adduct**	**RT (min)**
Hippuric acid-d5	1	183.081	$[M-H]^-$	0.76
Trans-Cinnamic acid-d5	2	152.075	$[M-H]^-$	2.95
Quercetin-d3	10	304.053	$[M-H]^-$	3.86
CUDA	1	339.265	$[M-H]^-$	5.71
Reserpine-d9	1	616.322	$[M-H]^-$	7.54
