# Supplementary material for: Neuroprotective Potential of Thinned Peaches Extracts Obtained by Pressurized Liquid Extraction after Different Drying Processes
Source: Foods. 2022 Aug 16;11(16):2464. doi: 10.3390/foods11162464 (PMC9407205; doi:10.3390/foods11162464)
Supplement: Supplementary file 1 [file foods-11-02464-s001.zip › Table S8.pdf]

Table S8. Abundancy of tentatively identified metabolites in thinned peach PLE extracts*.

**Retention time (min)**	**Metabolite name**	**FD**	**ND**	**HAD50**	**HAD70**	**HAD90**
0.811	3-*O*-coumaroylquinic acid	26959±5146^{b}	8904±451^{c}	44069±7761^{a}	21187±2638^{bc}	13668±5780^{bc}
1.780	4-*O*-caffeoylquinic acid	58510±3060^{a}	2837±321^{c}	6586±1535^{bc}	4352±529^{c}	8625±697^{b}
2.258	4-*O*-*p*-coumaroylquinic acid	7671±345^{a}	229±87^{c}	780±281^{c}	717±92^{c}	1968±193^{b}
1.072	5-*O*-feruloylquinic acid	6921±532^{bc}	3732±105^{c}	12588±3130^{a}	8922±81^{ab}	5958±517^{bc}
1.018	Chlorogenic acid	314649±12238^{a}	31346±3796^{c}	117777±39287^{b}	29635±913^{c}	27709±1671^{c}
0.238	Inositol 4-phosphate	6824±538^{b}	20581±1150^{a}	18410±1853^{a}	18143±248^{a}	18204±1274$_{a}$
0.572	Neochlorogenic acid	329205±23342^{a}	109394±9371^{b}	390871±63998^{a}	134957±3686^{b}	71992±4910^{b}
0.259	Quinic acid	565570±36717^{a}	455670±56509^{ab}	558890±57379^{a}	565188±35516^{a}	426868±38371^{b}
1.214	*Alpha hydroxy acid 1*	1912±257^{c}	4136±139^{a}	3547±444^{ab}	4125±266^{a}	3267±322^{b}
0.487	(2S)-2-(carbamoylamino)-4-(methylsulfanyl)butanoic acid	839±71^{b}	313±104^{c}	330±126^{c}	342±178^{c}	1352±163^{a}
0.415	3-Hydroxy-*L*-tyrosine	734±67^{bc}	536±64^{cd}	324±66^{d}	881±154^{b}	2223±159^{a}
0.996	Acetyl-leucine	212±112^{c}	8327±376^{a}	5961±1105^{b}	8551±507^{a}	5704±796^{b}
1.575	N-acetylphenylalanine	2896±226^{a}	1498±281^{b}	1252±298^{b}	1429±68^{b}	1429±88^{b}
4.260	3-Dimethylallyl-4-hydroxybenzoic acid	360±35^{c}	1096±88^{b}	287±33^{c}	1432±252^{ab}	1443±86^{a}
1.851	Benzoylmalic acid	620±145^{c}	25107±1002^{a}	14279±4151^{b}	14273±385^{b}	10896±641^{b}
0.279	Malate	271233±34343^{a}	189338±20589^{b}	154183±7145^{b}	195101±15045^{b}	183379±14156^{b}
0.631	*Carbohydrate 1*	263±64^{b}	1545±101^{a}	1544±150^{a}	223±43^{b}	341±31^{b}
0.328	*Carbohydrate 2*	894±83^{b}	427±95^{c}	408±105^{c}	994±78^{b}	2160±253^{a}
0.252	D-gluconic acid	21810±1224^{d}	200341±17106^{a}	78501±3415^{b}	43447±2834^{c}	10898±1591^{d}
0.262	D-glucose	37846±691^{a}	25838±2897^{b}	26434±1784^{b}	24842±1197^{b}	21684±2980^{b}
0.240	Maltotriose	2398±429^{a}	1447±107^{b}	2400±366^{a}	793±94^{b}	1213±116^{b}
0.243	Mannitol	78721±8861^{a}	35251±3175^{b}	37375±3024^{b}	35461±3487^{b}	38986±2912^{b}

1.146	Melilotoside	527±88^{c}	2820±287^{ab}	3013±585^{ab}	3642±374^{a}	2324±80^{b}
0.311	N-acetylmuramic acid	206±23^{c}	1064±123^{a}	902±233^{ab}	735±150^{ab}	575±28^{b}
1.308	Phenylethyl 2-glucoside	1277±183^{ab}	1191±97^{bc}	1717±335^{a}	930±152^{bc}	715±54^{c}
1.137	Prulaurasin	147528±5289^{a}	9619±770^{e}	36529±4909^{d}	99055±5136^{c}	114935±8616^{b}
1.421	Sayaendoside	2697±324^{d}	8531±608^{a}	6983±1089^{ab}	6699±156^{bc}	5257±591^{c}
0.239	Trehalose	47329±3662^{b}	34156±5026^{b}	130448±15950^{a}	11450±1431^{c}	12538±943^{c}
1.012	3,4-Dihydroxyacetophenone	3310±768^{a}	926±65^{b}	716±137^{b}	959±104^{b}	660±59^{b}
1.220	4-Hydroxybenzaldehyde	1489±90^{a}	1599±113^{a}	1600±338^{a}	1882±208^{a}	1583±310^{a}
2.175	Isopeonol	37±27^{c}	4179±264^{ab}	2655±2327^{abc}	4582±750^{a}	880±1461^{bc}
7.074	Cer 18:1;3O/24:0;(2OH)	320±37^{c}	4668±1444^{a}	2575±521^{b}	2897±383^{ab}	1613±396^{bc}
1.401	Gerberinside	18550±1177^{a}	26±28^{b}	10±0^{b}	1062±1822^{b}	1306±2245^{b}
0.342	Methylmalonic acid	10620±763^{a}	12230±1328^{a}	7670±503^{b}	6346±456^{b}	7074±597^{b}
0.802	Violaceic acid	10038±4040^{a}	13523±935^{a}	11647±2253^{a}	9048±1076^{a}	9118±3290^{a}
4.629	(9Z)-5,8,11-Trihydroxyoctadec-9-enoic acid	917±56^{d}	63927±1525^{a}	23874±5355^{c}	67461±2714^{a}	34743±1026^{b}
0.401	(2R)-2-(.beta.-*D*-glucopyranosyloxy)-2-phenylacetamide	2208±208^{c}	41054±4411^{a}	48764±4728^{a}	20046±1190^{b}	14869±844^{b}
1.900	Hexyl 6-*O*-pentopyranosylhexopyranoside	243±49^{c}	6595±526^{ab}	7548±1685^{a}	5535±204^{ab}	4656±170^{b}
0.247	Lactobionic acid	2406±103^{cd}	5613±227^{a}	4126±462^{b}	2515±201^{c}	1730±134^{d}
5.512	5,7-dihydroxyflavanone	52±11^{d}	2763±374^{a}	792±53^{c}	2159±283^{ab}	1591±175^{b}
4.379	Naringenin	521±139^{c}	1903±95^{a}	837±110^{c}	1493±101^{b}	1468±193^{b}
4.838	Isorhamnetin	418±29^{c}	1330±127^{ab}	1101±196^{b}	1495±194^{a}	1240±59^{ab}
4.659	Kaempferol	356±20^{b}	1737±65^{a}	1619±371^{a}	1720±212^{a}	1425±225^{a}
4.016	*Flavonoid glycoside 1*	834±146^{d}	3501±383^{ab}	4572±804^{a}	2520±128^{bc}	1886±89^{cd}
1.826	*Flavonoid glycoside 2*	867±81^{c}	1839±9^{b}	3301±762^{a}	645±42^{c}	549±100^{c}
2.334	Eriodictyol-7-*O*-glucoside	518±35^{b}	2670±87^{b}	12357±3155^{a}	710±83^{b}	620±139^{b}
2.371	Hyperin	1691±193^{c}	3942±88^{b}	6903±1707^{a}	1309±63^{c}	1034±25^{c}
2.723	Ideain	1679±156^{c}	8571±1073^{a}	7597±1123^{ab}	6451±225^{b}	5931±515^{b}

2.862	Isorhamnetin-3-*O*-glucoside	1470±213[c]	5849±401[a]	5662±1108[a]	3632±128[b]	3075±28[b]
2.768	Isorhamnetin-3-*O*-rutinoside	5292±556[c]	24796±1852[a]	25003±5244[a]	16552±408[b]	14220±705[b]
2.632	Kaempferol-3-*O*-rutinoside	3632±206[d]	28694±2306[a]	26010±5337[ab]	20297±697[bc]	16797±1291[c]
2.786	Naringenin-7-*O*-glucoside	2052±96[c]	6070±347[b]	9501±2132[a]	2708±179[c]	2691±120[c]
2.302	Quercetin-3-*O*-rutinoside	1619±188[c]	4013±168[b]	7267±1615[a]	1290±135[c]	1046±124[c]
2.575	Quercitrin	570±70[b]	2644±121[a]	2452±777[a]	1942±396[a]	1679±224[a]
2.107	Rutin	235±181[c]	1221±111[a]	1080±301[ab]	853±89[ab]	749±60[b]
0.480	2-(1-hydroxyethyl)-4-(2-hydroxypropyl)-2H-furan-5-one	1558±247[bc]	361±61[c]	236±80[c]	2672±282[b]	21649±1252[a]
6.049	2-Linoleoyllysophosphatidylcholine	6977±878[a]	940±187[c]	2685±319[b]	1294±20[c]	2695±279[b]
5.948	LPC 18:3	3171±301[a]	315±36[c]	1210±248[b]	403±70[c]	1065±26[b]
6.802	Phosphatidylcholine(16:0/18:2w6)	1863±1921[a]	698±271[b]	743±191[b]	825±494[b]	494±183[bc]
5.989	LPG 18:3	961±84[a]	338±57[cd]	517±66[bc]	284±22[d]	706±146[b]
5.643	*Glycerophosphoinositol 1*	3258±410[a]	1034±84[bc]	3436±534[a]	773±141[c]	1749±141[b]
5.783	DGMG 18:3	2920±185[b]	1025±113[c]	2503±459[b]	7952±343[a]	8663±673[a]
6.669	MGDG 18:3/18:3	19450±1220[a]	550±95[d]	2607±499[cd]	4538±641[bc]	6605±1242[b]
0.981	Caffeic acid	8635±245[a]	791±140[c]	2170±466[b]	901±77[c]	984±257[c]
1.857	Isoferulic acid	2553±729[a]	244±38[b]	309±103[b]	194±3[b]	247±52[b]
1.724	4-Methyldaphnetin	2132±255[a]	1146±69[b]	1056±350[b]	2204±243[a]	2324±438[a]
1.105	Tryptophan	372±31[c]	8165±832[a]	9563±784[a]	3995±380[b]	705±116[c]
5.976	9-Hydroxy-10E,12Z-octadecadienoic acid	381±113[d]	26555±2249[a]	4944±1039[bc]	7790±388[b]	3134±363[cd]
4.228	Corchorifatty acid F	981±121[e]	36752±1478[b]	12624±1981[d]	46556±794[a]	22139±1317[c]
1.576	Coniferyl aldehyde	3810±1066[a]	162±46[b]	241±96[b]	213±62[b]	247±18[b]
5.460	Gingerol	5533±137[a]	53±4[b]	86±44[b]	47±16[b]	71±44[b]
1.377	DL-3-phenyllactic acid	5034±424[a]	1722±121[b]	1593±603[b]	2064±161[b]	2065±244[b]
0.334	Adenine	1581±435[c]	4150±438[a]	2759±71[b]	3772±481[a]	2331±239[bc]
0.390	3-Hydroxypicolinic acid	6299±1499[b]	11237±1179[a]	12459±2141[a]	7303±239[b]	4815±279[c]

1.443	*Terpene glycoside 1*	48±18^{d}	15581±1060^{a}	10596±1803^{b}	3070±111^{c}	3976±205^{c}
0.453	Theviridoside	1420±81^{a}	202±61^{b}	253±130^{b}	254±81^{b}	405±58^{b}
5.685	*Triterpenoid 1*	298±27^{d}	6660±613^{a}	3787±579^{b}	3091±358^{bc}	2579±163^{c}
5.803	*Triterpenoid 2*	186±21^{c}	4688±302^{a}	1722±288^{b}	1220±216^{b}	1511±156^{b}
5.552	*Triterpenoid 3*	146±39^{d}	1389±65^{a}	742±195^{bc}	880±36^{b}	557±55^{c}
5.980	Hederagenin	1556±237^{c}	5198±683^{a}	3627±551^{b}	1324±203^{c}	2268±443^{c}
6.225	Oleanoic acid	4626±217^{a}	4623±406^{a}	3591±486^{b}	1390±92^{d}	2519±502^{c}

* Different letters in the same row indicate significant differences between samples after ANOVA with Tukey's Post-hoc (p-value < 0.05).
