# Supplementary figures and images for: Neuroprotective Potential of Thinned Peaches Extracts Obtained by Pressurized Liquid Extraction after Different Drying Processes
Source: Foods. 2022 Aug 16;11(16):2464. doi: 10.3390/foods11162464 (PMC9407205; doi:10.3390/foods11162464)

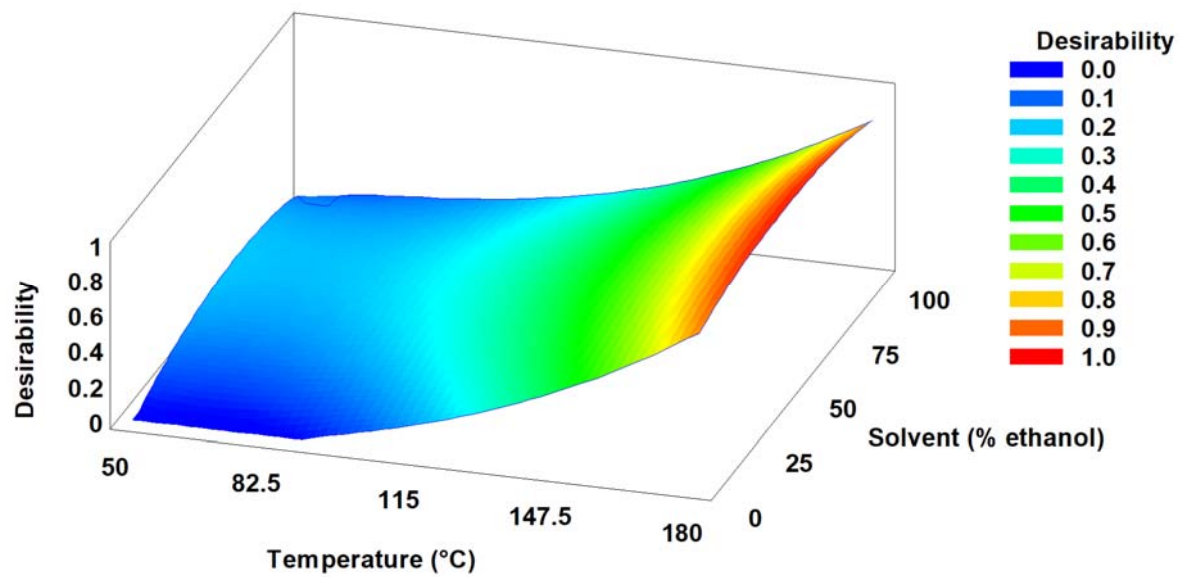

**Figure S2.** Desirability response surface to optimize all response variables.

Supplement: Supplementary file 1 [file foods-11-02464-s001.zip › Figure S2.pdf]
